# A Kinetic-Based Model of Radiation-Induced Intercellular Signalling

**DOI:** 10.1371/journal.pone.0054526

**Published:** 2013-01-22

**Authors:** Stephen J. McMahon, Karl T. Butterworth, Colman Trainor, Conor K. McGarry, Joe M. O’Sullivan, Giuseppe Schettino, Alan R. Hounsell, Kevin M. Prise

**Affiliations:** 1 Centre for Cancer Research and Cell Biology, Queen’s University Belfast, Belfast, Northern Ireland, United Kingdom; 2 Radiotherapy Physics, Northern Ireland Cancer Centre, Belfast Health and Social Care Trust, Northern Ireland, United Kingdom; 3 Clinical Oncology, Northern Ireland Cancer Centre, Belfast Health and Social Care Trust, Belfast, Northern Ireland, United Kingdom; National Taiwan University, Taiwan

## Abstract

It is now widely accepted that intercellular communication can cause significant variations in cellular responses to genotoxic stress. The radiation-induced bystander effect is a prime example of this effect, where cells shielded from radiation exposure see a significant reduction in survival when cultured with irradiated cells. However, there is a lack of robust, quantitative models of this effect which are widely applicable. In this work, we present a novel mathematical model of radiation-induced intercellular signalling which incorporates signal production and response kinetics together with the effects of direct irradiation, and test it against published data sets, including modulated field exposures. This model suggests that these so-called “bystander” effects play a significant role in determining cellular survival, even in directly irradiated populations, meaning that the inclusion of intercellular communication may be essential to produce robust models of radio-biological outcomes in clinically relevant *in vivo* situations.

## Introduction

The central dogma of radiation biology – that the biological effects of radiation are due to DNA damage resulting from ionisations caused by the incident radiation – has been extensively challenged in recent years. It is clear now that while direct DNA damage does play an important role in cellular survival, a variety of indirect processes (that is, those affecting cells which are not directly irradiated) also significantly impact on cellular responses to radiation [1]. This radiation-induced “bystander” effect, where cells not exposed to ionising radiation experience DNA damage and mutations as a result of communication with irradiated cells, has been demonstrated for cells in direct contact, sharing culture media, and when media from irradiated cells is transferred to unirradiated cells [2]–[7].

However, despite the apparent ubiquity of these effects, they are not typically incorporated into mathematical descriptions of the effects of ionising radiation, either in the analysis of *in vitro* laboratory experiments or epidemiological *in vivo* data. For example, radiotherapy treatments for cancer are typically planned based on the assumption that the probability of killing tumour cells at a given point is a function solely of the dose delivered to that point [8]. While this was not a significant factor in the past, where relatively uniform radiation fields were used, the use of increasingly complex spatially modulated treatment fields, through delivery techniques such as Intensity Modulated Radiation Therapy and charged particles, may lead to indirect effects becoming increasingly significant [9]. Similarly, extrapolation of the risks associated with low doses from high dose data may be significantly complicated if a small portion of irradiated cells were able to lead to adverse effects in large numbers of neighbouring cells [10].

One of the major challenges preventing incorporation of these effects in biological models is the lack of robust mathematical descriptions of the underlying processes. Numerous models have been developed to describe intercellular signalling following radiation [11]–[18], but recent work investigating the effects of modulated X-ray fields is inconsistent with many of their assumptions or predictions. These include:

– That there is a separation between “hit” and “bystander” cells, such that only un-hit cells suffer signalling-induced damage [11]–[13], [17]. While many models were developed with reference to charged particle studies, where this distinction is meaningful, in X-ray exposures the vast majority of cells see some ionising events, even at very low doses. Additionally, these studies showed significant signalling-induced killing, even when shielded populations were exposed to one Gray or more [6], [7], indicating that direct exposure to radiation does not mitigate signalling effects.
– That signal levels and corresponding responses are proportional to the number of irradiated cells [11]–[13], [15]–[18]. In modulated field and some media transfer studies, a threshold effect is observed, with no effect when small numbers of cells are irradiated, but with a nearly constant effect above this threshold.
– That these effects saturate at low doses, either because of the above assumptions or because irradiated cells produce fixed levels of signal, independent of dose [11]–[15]. While many media transfer experiments show saturation [19], recent studies of modulated field exposures have shown changes in signal levels up to doses of 8 Gray in irradiated populations [4], [6].
– That even very low signal concentrations can cause a response [11]–[18]. By contrast, studies of media dilution and modulated fields have shown that there are clear thresholds before genotoxic responses are triggered [7], [20].

Finally, many models make largely empirical links between radiation exposure and the consequences of intercellular communication, which makes comparisons between different experimental protocols and possible *in vivo* effects challenging. As a result, it is not possible to use many published models to describe the full variety of experimental investigations of non-targeted effects which are seen in the literature, suggesting the need for a more generally applicable description of these effects.

Our group recently presented a model [21] describing the response of cells to modulated radiation exposures, incorporating signalling effects, which was found to accurately reflect experimental observations. However, as in many of the above models, this used an empirically fitted constant to describe the relationship between delivered dose and signal levels, reducing its general applicability. In this work, we generalise this model by introducing a mechanistic model of signalling and response, to address many of the above discrepancies. The key assumptions of this model can be summarised as follows:

– Irradiated cells generate signal for an extended time period proportional to the delivered dose, regulated to reach some local equilibrium concentration;
– Exposure to this signal above a certain threshold concentration can lead to a damaging response in cells, with a probability related to the time the cell is exposed to the signal above this threshold;
– This response is binary, with responding cells experiencing a characteristic level of cell damage and non-responding cells seeing no damage;
– Signalling-induced damage can occur in both hit and non-hit cells, and is additive to other sources of damage, such as that resulting from direct irradiation.

**Figure 1:**
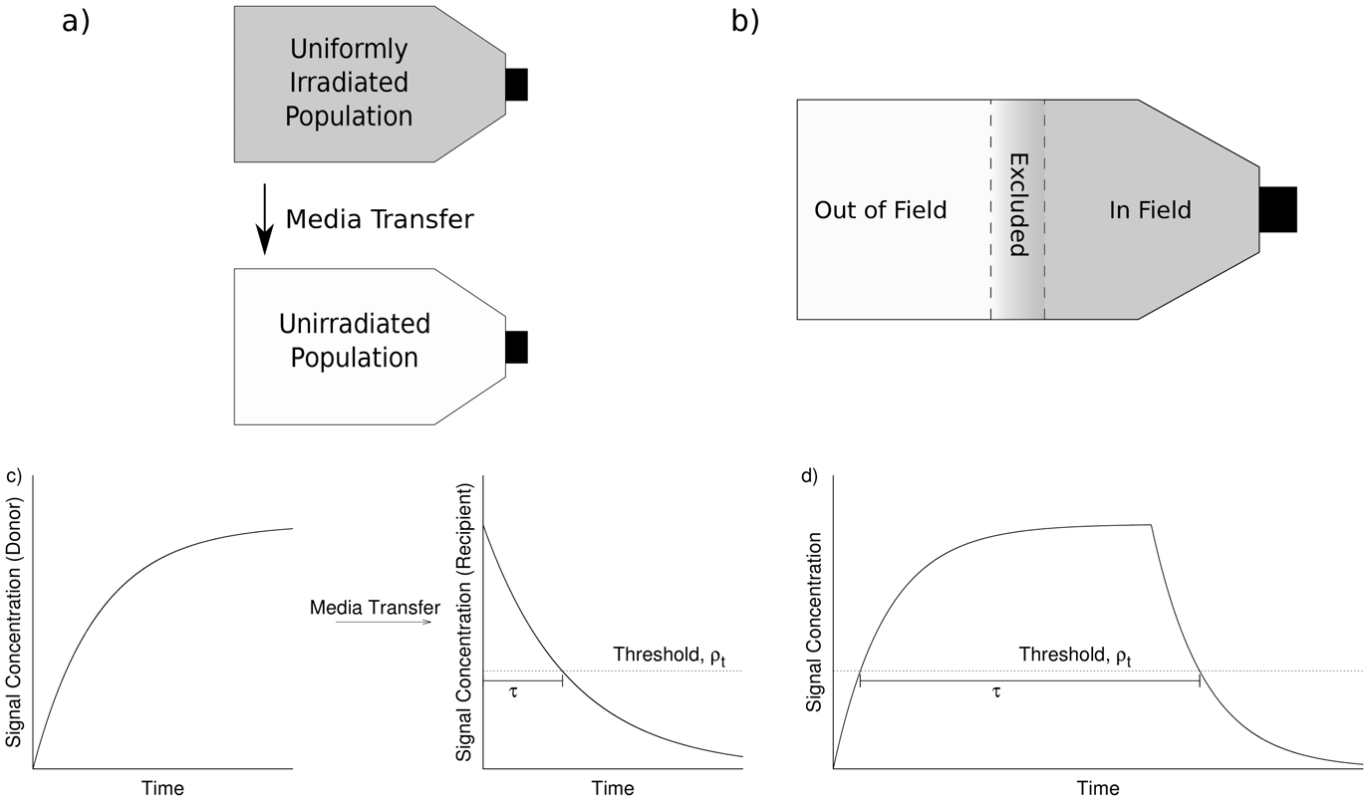
Illustration of common experiments investigating radiation-induced signalling. Top: Schematic illustration of experimental approaches modelled in this paper. Intercellular communication is investigating by transferring media from irradiated cells to unirradiated cells (media transfer, a) or by exposing cell populations to spatially varying doses (modulated field, b), and measuring changes in survival in populations not exposed to radiation. Bottom: Illustration of signal kinetics expected in each system. Probability of response is governed by the time t_exp_, for which the signal is above the threshold. Due to prolonged signal production, this is significantly longer in modulated field exposures (d) than in media-transfer experiments (c).

A schematic illustration of the kinetics of the signal in two typical experimental set-ups is shown in Figure 1, and a mathematical formulation of these kinetics is presented in the methods. This model was then tested by fitting it to a series of experimental conditions, as described in the results.

## Models and Methods

### Signal Production

While several potential signalling pathways and molecules have been implicated in intercellular communication following radiation exposure, clear experimental evidence for role of different factors is lacking, so in this work it is modelled as a single concentration, ρ, which is spatially- and temporally-dependent.

In this model, which focuses on acute exposures, cells begin to produce these signals immediately following exposure to ionising radiation, and continue to do so for a time proportional to the delivered dose - that is, for a time 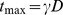, where *D* is the dose delivered to the cell and γ is a constant, characteristic of the cell-line.

While actively signalling, cells seek to maintain a local concentration of ρ_max_. This is modelled as the signal production decreasing linearly as ρ increases – given by 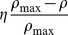, where η is the rate of signal production by a cell if the local signal concentration is 0, depending on both cell line and culture media. Biologically, this could be interpreted as the signal being involved in negative feedback of some kind – as is seen in some radiation-induced pathways and stress responses [22], [23]. This signal is assumed to decay over time, modelled as a simple exponential decay with a rate constant of λ. Based on evidence that these signals are common across different cell-lines [24], it is assumed that this constant is independent of the source cell type.

If the signal spreads out via diffusion (or other reasonably spatially uniform processes), then the signal concentration in the system evolves, in the absence of any new sources, according to
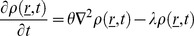
(1)where **r** is a spatial position, *θ* is the diffusion coefficient and 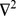 is the Laplacian operator. Thus, in general, a given signal will tend to spatially equilibrate as it decays.

Irradiated cells can then be represented as a series of point sources of signal. However, even for a single cell, equation 1 does not have a general analytic solution, due to the complex interplay between the rates of signal production, decay, and diffusion.

By contrast, numerical solutions of equation 1 are straightforward – as outlined in the models and methods S1– but often prohibitively time-consuming. However, in most *in vitro* studies of these effects, little spatial variation is observed [25], suggesting that the rate of diffusion is much greater than the rate of signal production. As a result, it is reasonable to assume that ρ is uniform, allowing for the reformulation of equation 1 as
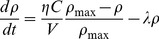
(2)where C is the number of signalling cells and V is the total media volume, which appears because the signal quantity 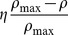 produced by each individual cell is taken to rapidly spread throughout the entire volume.

For a number of cells, C_I_, irradiated at a time t = 0, this can be exactly solved to give:
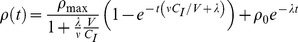
(3)where ρ_0_ is the concentration at t = 0, and the substitution 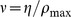 has been made for brevity. This simplified description largely reflects the kinetics of the full numerical modelling of the system (as shown in figure S1).

However, some quantitative discrepancies between this analytic solution and the numerical analysis exist. Most significantly, while average concentrations are a useful description at long times, there is some heterogeneity at early times, particularly in the vicinity of signalling cells, which leads to lower rates of signal production.

As a result, the rate at which the signal approaches equilibrium following irradiation is significantly slower than 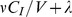 predicted above. Instead, as can be seen in figure S2, the rate is not substantially larger than λ for cell concentrations less than 10,000 cells per mL, and remains on this order, saturating at less than 2.5λ.

To account for this, a simplification has been made in this model, fixing the exponential rate term as λ, regardless of the number of cells which are signalling, giving
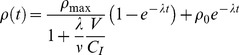
(4)


This relationship retains the overall scaling of equation 3, while also providing much better agreement at early times. While this introduces a slight discrepancy at extremely high cell densities, it is minor in the data sets considered here.

In the above case, cells exposed to a dose D cease signalling after a time γD. After this, equation 4 reduces to a simple decay, characterised as 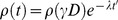, where ρ(γD) is the concentration at t = γD, and t′ = t-γD. An illustration of the resulting kinetics can be seen in Figure 1.

More generally, there are multiple populations of cells exposed to different doses. In the case of two cell populations of number C_1_ and C_2,_ exposed to doses D_1_ and D_2_ (D_1_<D_2_) respectively, the signal concentration will initially evolve according to equation 4, with C_I_ = C_1_+C_2_. At a time γD_1_, the cell population C_1_ will cease signalling, and the signal is then given by
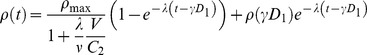
(5)


Finally, at time γD_2_, the remaining cells cease signalling, and the signal decays as a simple exponential. This can be extended, in a similar fashion, over any number of sub-populations exposed to different doses to describe the signal kinetics following an arbitrary radiation pattern in these *in vitro* radiation exposures.

It should be noted that while the assumption of spatial homogeneity has been made above to facilitate efficient fitting to the data sets, numerical modelling of intercellular signalling, as outlined in the supporting information, is also viable based on the same fundamental assumptions, and leads to similar results for these systems. These numerical models also allow for descriptions of signalling in systems where signal propagation is much slower, such as in the skin model described below.

### Response to Radiation-Induced Signalling

The response of cells to these signals is a binary event – that is, cells either respond and see (on average) a fixed level of damage, or do not and see no effect [26], [27]. The exact mechanism by which this DNA damage is induced is not yet fully elucidated, although it is believed that membrane-mediated signalling pathways and elevated levels of oxidative stress in recipient cells plays a role [27], [28].

In our previous work [21], this effect was studied for a single irradiation geometry, and was characterised as a simple exponential dependence, 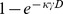, where κγ was a fitted parameter and D was the in-field dose, but this is obviously insufficient to describe the full range of experiments considered here.

In this work, the probability of a response is predicted based on the signal kinetics outlined above. Specifically, based on evidence that these signals have a threshold below which no effect is observed [20], we propose that the probability that a cell responds to these signals scales with the total time that it is exposed to a signal concentration above a certain threshold, ρ_t._ The exponential dependence is retained, giving
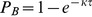
(6)where P_B_ is the probability of a cell experiencing a stress response due to radiation-induced signalling, and τ is the total time where ρ>ρ_t_. This period is illustrated in Figure 1.

As noted above, cells which respond to these signals experience genotoxic stress, which can potentially lead to the induction of DNA damage, mutation and cell death [26], [27], [29], [30]. This is modelled either as a simple probability of mutation induction or cell death or, where direct and intercellular signalling can potentially combine, a previously published model of radiation damage is used. This is briefly reviewed below for completeness.

### DNA Damage Model

This model was originally developed for a computational model of cellular response to ionising radiation [31], and was extended to include effects of intercellular communication in a previous work [21].

In this model, DNA damage in cells (either resulting from direct radiation, or as a consequence of genotoxic stresses which are triggered by intercellular communication) is represented by a number of “hits”, which can be viewed as potentially lethal events, such as complex or unrepaired double-strand breaks.

Hits from ionising radiation are generated by sampling a Poisson distribution, with a mean proportional to the delivered dose. Indirect damage due to intercellular signalling is represented as additional hits, generated by sampling from a Poisson distribution with a mean of H_B_, which is a characteristic of the cell line.

Depending on the level of damage, cells may then either die immediately (cells which accumulate ≥5 hits), experience arrest in the G1 phase (≥3 hits), or, in the special case where cells were irradiated in the G2 phase, they will be arrested following small amounts of damage (1 hit).

More detail on the rationale and development of this model can be found in previous publications [21], [31]. An example implementation of this model, applied to a half-field irradiation (such as that of Butterworth et al) is presented in example code S1.

### Data Fitting

The above models of signal production and response allow for predictions to be made of the probability of cells experiencing damage due to intercellular communication, as well as for the more general situations which also incorporate direct irradiation.

Responses to intercellular communication are characterised by the parameters ν, ρ_max_, λ, γ, ρ_t_, and κ. In most data sets, there is insufficient experimental data to uniquely fit all of these parameters. To address this, it has been assumed in this work that the signal decay rate, λ and the signal threshold, ρ_t_/ρ_max,_ are constant across all experiments. A single fit was carried out over all media transfer and modulated field experiments, fitting the signal kinetic parameters plus cell- and experiment-specific response parameters (e.g. probability of cell death or mutation frequency in responding cells) to the data set, by χ^2^ minimisation.

Effects in the skin model were fit separately (as numerical simulations of signal propagation were prohibitively time-consuming as part of the above ensemble fit), again by χ^2^ minimisation.

## Results

### Media Transfer Experiments

Some of the first evidence for the effects of intercellular communication following irradiation were media transfer experiments [19]. In these experiments, a population of cells is uniformly irradiated and incubated for a time to allow for signal generation. The medium is then removed from these donor cells, filtered and added to a recipient cell population. These recipient cells see an increase in DNA damage, genomic instability and cell death, compared to cells grown in media taken from unirradiated cells. The resulting signalling kinetics are schematically illustrated in Figure 1.

**Figure 2:**
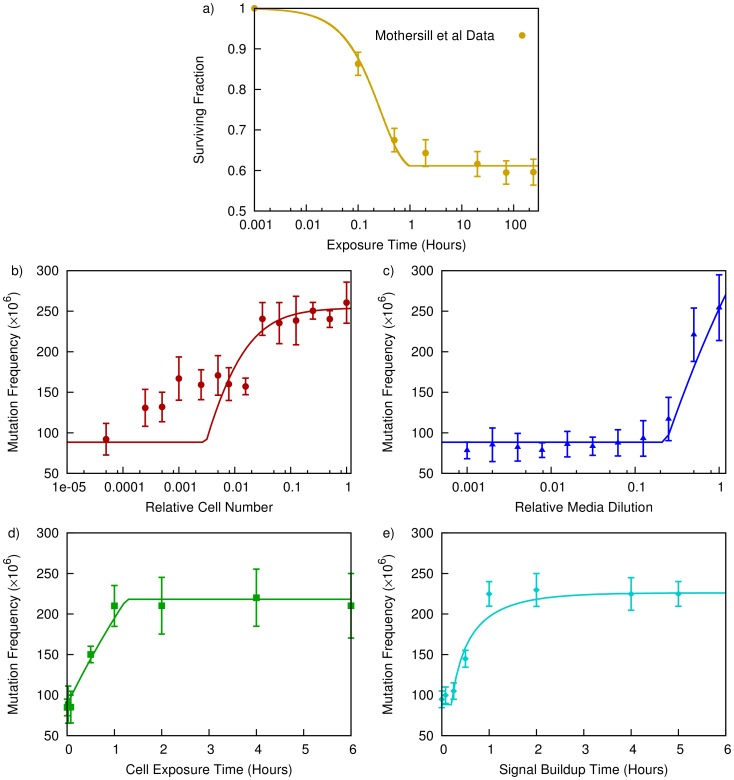
Comparison of model fits to media transfer experiments of Mothersill et al [19] and Zhang et al [32]. In Mothersill et al (a), the model (solid line) is able to reflect the onset of signalling-induced cell killing seen in a media-transfer experiment as a function of exposure time (circle). For Zhang et al (b-e), the model’s predictions (lines) are compared to observed mutation rates when the number of cells exposed to ionising radiation (b), the level of media dilution before media transfer (c), the amount of time the recipient cells are exposed to the donor media (d) or the amount of time before the donor media was harvested (e) was varied. Good agreement with the overall trends is found in all cases, with the exception of the small plateau in cell number dilution, which suggests some additional complexity in signal production at reduced cell densities.

Two media transfer experiments are considered in this work. Firstly, one of the earliest demonstrations of the effects of radiation-induced signalling, made by Mothersill et al [2]. Here, a population of 20,000 HaCat human keratinocyte cells were exposed to 5 Gy of radiation, and incubated for 1 hour. The treated media was added to a population of recipient cells for times varying from 6 minutes to 240 hours. Following this exposure, the clonogenic survival of the recipient cells was measured. These results are plotted in Figure 2a, showing an initial drop in survival with exposure time, which saturates after approximately two hours.

Secondly, the data set of Zhang et al [32] was investigated, as it offered a more robust of test the parameters in this model. Here, the number of mutations induced by media-transfer mediated signalling was investigated in WTK1 lymphoblastoid cells. In the basic form of this experiment, 2.5×10^6^ WTK1 cells were suspended in 5 mL of media, and irradiated with a dose of 2 Gy. The cells were then incubated for 2 hours, after which the media was transferred to a recipient population for 24 hours. Following this, frequencies of mutations in the recipient cells were determined.

Four parameters were then varied from this basic experimental condition to determine their effects on mutation frequencies induced by the intercellular signalling: Cell density, media dilution, the time for which the recipient cells were exposed to the media, and signal incubation time. These results are plotted in Figure 2 b-e, respectively. Clear variations are seen with all these of these variables, with mutation frequency falling rapidly with signal dilution and reducing cell density, and showing a build-up time on the order of an hour for both incubation and exposure.

In both of the above cases, the transferred medium contains a signal of ρ_0_, given by equation 4 as:
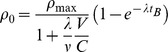
where t_B_ is the amount of time for which the signal is allowed to build up. Once added to the recipient cells, the signal decays according to 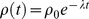, so the maximum time the signal will remain above the response threshold is given by 
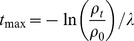
. This means that the amount of time the cells are exposed to a signal above the threshold is given by 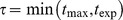, where t_exp_ is the amount of time recipient cells were exposed to the media from the irradiated cells. Finally, the probability of a cell responding to the signal is given by 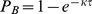. This response probability is common to both of the above data sets. However, different endpoints were used in each experiment.

In Mothersill et al, cell killing was used as an endpoint. It is assumed that cells which respond to the intercellular signalling have a fixed probability of cell death, and the total survival probability can be expressed as 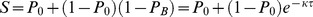 where S is the fraction of surviving cells, and P_0_ is the probability that a cell survives following response to these signals. This predicted curve is plotted alongside the data in Figure 2a as a solid line.

Zhang et al used mutation frequency as an endpoint. As above, a fixed mutation probability MF_B_, is associated with response to the signalling process. Thus, the total mutation probability can be expressed as 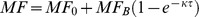, where MF_0_ is the base mutation frequency. The model’s predictions for the variation in mutation frequency with each of the variables under consideration are plotted as solid lines in Figure 2 b-e.

The fitted model parameters are presented in Tables 1 and 2, showing signal kinetic and cellular response parameters, respectively. It should be noted that, in all cases, the fits are independent of the absolute value of ρ_max_ or ρ_t_, depending only on their ratio.

**Table 1:** Signal Kinetic Parameters.

	*Common Parameters*						
λ	0.019±0.002						
ρ_t_/ρ_max_	0.21±0.02						
	*HaCat*	*WTK-1*	*AGO-1522*	*DU145*	*H460*	*MM576*	*Skin Model*
γ (min Gy^−1^)	–	–	140±10	61±20	132±30	120±40	180±80
κ (min^−1^)	0.061±0.01	0.0040±0.001	0.0027±0.0007	0.0028±0.001	0.0029±0.001	<0.0024	0.006±0.004
ν (min^−1^)	(5.8±2)×10^−6^	(8±1)×10^−6^	(1.1±0.3)×10^−4^	(1.1±0.4)×10^−4^	(8±3)×10^−5^	(1.3±0.4)×10^−4^	–
γκ (Gy^−1^)	–	–	0.38±0.035	0.17±0.05	0.38±0.08	0.29±0.09	1.2±0.2

Table 1: Parameters describing cellular signal kinetics modelled for the experiments described in the text. Parameters are best-fits obtained by χ^2^ minimisation, quoted with 66% confidence intervals. λ and ρ_t_/ρ_max_ are common to all data sets, while other parameters are cell-line specific.

**Table 2:** Response Parameters.

	*Mothersill et al*			
*P_0_*	0.6±0.4			
	*Zhang et al*			
*MF_0_*	(88±3)×10^−6^			
*MF_B_*	500×10^−6^ (250×10^−6^, ∞)			
	*Butterworth et al; Suchowerska et al*			
	*AGO-1522*	*DU145*	*H460*	*MM576*
*H_B_*	2.1±0.2	3.0±0.4	3.7 (2.7, ∞)	2.1 (1.8, ∞)
*Hits/Gy*	0.96±0.03	0.78±0.06	0.78±0.1	0.46±0.01
	*Belyakov et al*			
*Apoptotic Response*	3.6±0.5%			

Table 2: Parameters describing cellular responses to radiation-induced signalling and ionising radiation. Parameters are best-fits obtained by χ^2^ minimisation, quoted with 66% confidence intervals.

Good agreement is seen with both experiments, across the majority of the parameters considered. Some disagreement is seen in the Zhang et al data at moderate cell dilutions, but this may be due to a breakdown in the assumptions of homogeneity and uniformity used to facilitate fitting this data.

### Modulated Field Exposures

While media transfer experiments clearly demonstrate the effects of radiation-induced signalling, they are very different to *in vivo* situations, where cell populations necessarily remain in contact for extended periods. This is partially addressed in *modulated field exposures*. In these experiments (e.g. [4], [6]), a flask of cells is irradiated by a non-uniform field, and cells exposed to high and low doses share media for an extended period. Following this, cell survival or DNA damage is measured in different areas of the flask, allowing for a quantification of direct and indirect effects. Because of the prolonged contact, out-of-field cells are exposed to signals from irradiated cells for a longer period than in media transfer experiments, as illustrated in Figure 1.

Two sets of modulated field exposure experiments are studied here. Firstly, a series of experiments from Butterworth et al [7], which investigated the effects of modulated radiation fields on AGO-1522 and DU-145 cells. These cells were plated in T80 flasks, and exposed to modulated radiation fields, as illustrated in Figure 1, with a high-dose “in-field” region and an “out-of-field” region where the dose was reduced by introducing attenuating filters. A clear contribution from intercellular communication was seen, with significantly lower survival in the low-dose region than would be predicted from the dose delivered to that region alone.

**Figure 3:**
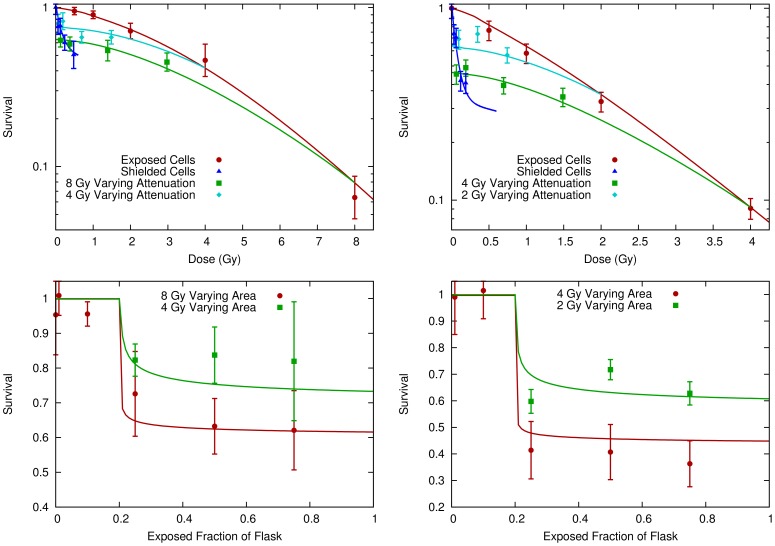
Comparison of model (lines) to modulated field survival data (points) from Butterworth et al [7]. Here, DU145 (left) or AGO-1522 (right) cells were irradiated using a stepped dose field. All points are cell survival in a region against the dose delivered to that region. Top: Effect of varying delivered doses. Varying doses were delivered to directly exposed cells (red circles) while the out-of-field transmission was held fixed at 3% by shielding the cells (blue triangles). In addition, experiments were carried out where the in-field dose was constant and the out-of-field dose was varied by changing the level of shielding (light blue diamonds, green squares). Bottom: Effect of varying area in-field was investigated by holding the dose and attenuation constant, and varying the fraction of the flask under the shielding.

Figure 3 presents the effects of varying of several experimental parameters on survival in this scenario, including the dose delivered in-field; the degree of attenuation in the out-of-field region; and the number of cells irradiated.

To investigate the influence of in-field dose, a range of doses were delivered to the flask while 50% of the flask was shielded with an alloy that transmitted 3% of the dose seen in the exposed region. The effect of out-of-field dose was tested by holding the in-field dose fixed and varying the degree of shielding, for transmissions varying from 1.6 to 37.2%. In both cases, it can be seen that there is a significant decrease in survival for cells which share media with cells exposed to a higher dose, with survival approaching that of uniformly exposed cells as the degree of transmission is increased.

Finally, the effect of the fraction of cells irradiated was investigated by holding both the dose and transmission fixed, and varying the portion of the flask that was covered by the shielding, which shows a clear cell number threshold in the out-of-field effect, below which no effect is observed.

**Figure 4:**
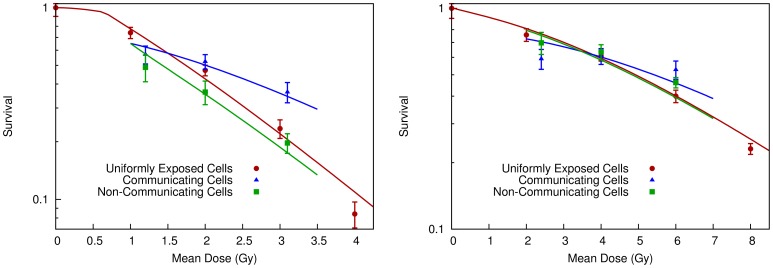
Comparison of model (lines) to modulated field survival data (points) from Suchowerska et al [4]. Here, H460 (left) or MM576 (right) cells were exposed either uniformly (red circles) or to a modulated radiation field created by a wedge filter. Modulated field exposures were further sub-divided into conditions where cells at all doses were free to communicate in a T175 flask (blue triangles), or where communication was inhibited between different dose levels by carrying out the irradiation in multiple smaller T25 flasks (green squares). Cell survival is taken as the average survival in a given small flask or corresponding region of the larger flask, and plotted against the average dose in that region.

A second study which made use of modulated fields is that of Suchowerska et al [4]. In this work, NCI-H460 and MM576 cells were exposed to either uniform irradiation or to a dose gradient created by a 60° wedge filter. These exposures were carried out either in one T175 flask, or multiple smaller T25 flasks. The contribution of intercellular signalling was evaluated by comparing survival in regions of T175 flasks to that in T25 flasks which saw equal doses but where communication between low- and high-dose regions was inhibited. The resulting survival curves are shown in Figure 4, showing a variation in survival which can be attributed to variations in communication between cells seeing different doses.

In both scenarios, shielded cells experience longer exposure times, τ, to signalling from cells exposed to high doses than seen in media-transfer experiments, as exposure time is no longer dominated by signal decay. Instead, it is primarily determined by the total time γD for which the signal is produced. In the limiting condition where 1/λ is small relative to γD, this leads to the 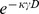 dependence in our earlier model.

To characterise the total time cells are exposed to signals above the response threshold, the evolution of the signal is calculated according to equations 4 and 5, and the times when the signal concentration first rises above the threshold level (t_min_) and when it falls below the threshold value (t_max_) are calculated from these expressions. Then, the probability of a cell experiencing a damaging response is once again given by 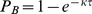, with 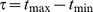. Signal kinetics were modelled by fitting ν, λ, γ, and ρ_t_ as above; as well as H_B_, the number of “hits” induced in responding cells. The direct effects of radiation are included by fitting the number of hits induced per Gy of radiation in directly exposed cells. Once again, the solid lines in Figures 3 and 4 show the fitted model predictions, based on the fitting parameters presented in Tables 1 and 2.

Once again, agreement is seen between the model and observed data for all conditions. Over all of the data fitted in Figures 2 through 4, the reduced χ^2^ sum is 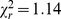, corresponding to a p value of 0.28 for the observed data sets resulting from the predicted distributions, suggesting it is a good reproduction of the underlying behaviours.

### Experimental Validation

One of the main assumptions of this model is that responses to intercellular signalling following radiation exposure build up over time due to a prolonged exposure to signals, rather than due to total signal absorbed as has been suggested in other models. While this is supported by the trends observed in the above experiments, it is also possible to measure this effect directly, by incubating cells together for some period following a modulated exposure and then separating them.

To study this, DU-145 cells were densely seeded in a P90 dish (8×10^5^ cells per dish, or 12,500 cells per cm^2^), and half of the cells were exposed to 8 Gy of radiation. These cells were incubated together for times ranging from 0 to 24 hours, then separated into irradiated and non-irradiated populations in new flasks.

**Figure 5:**
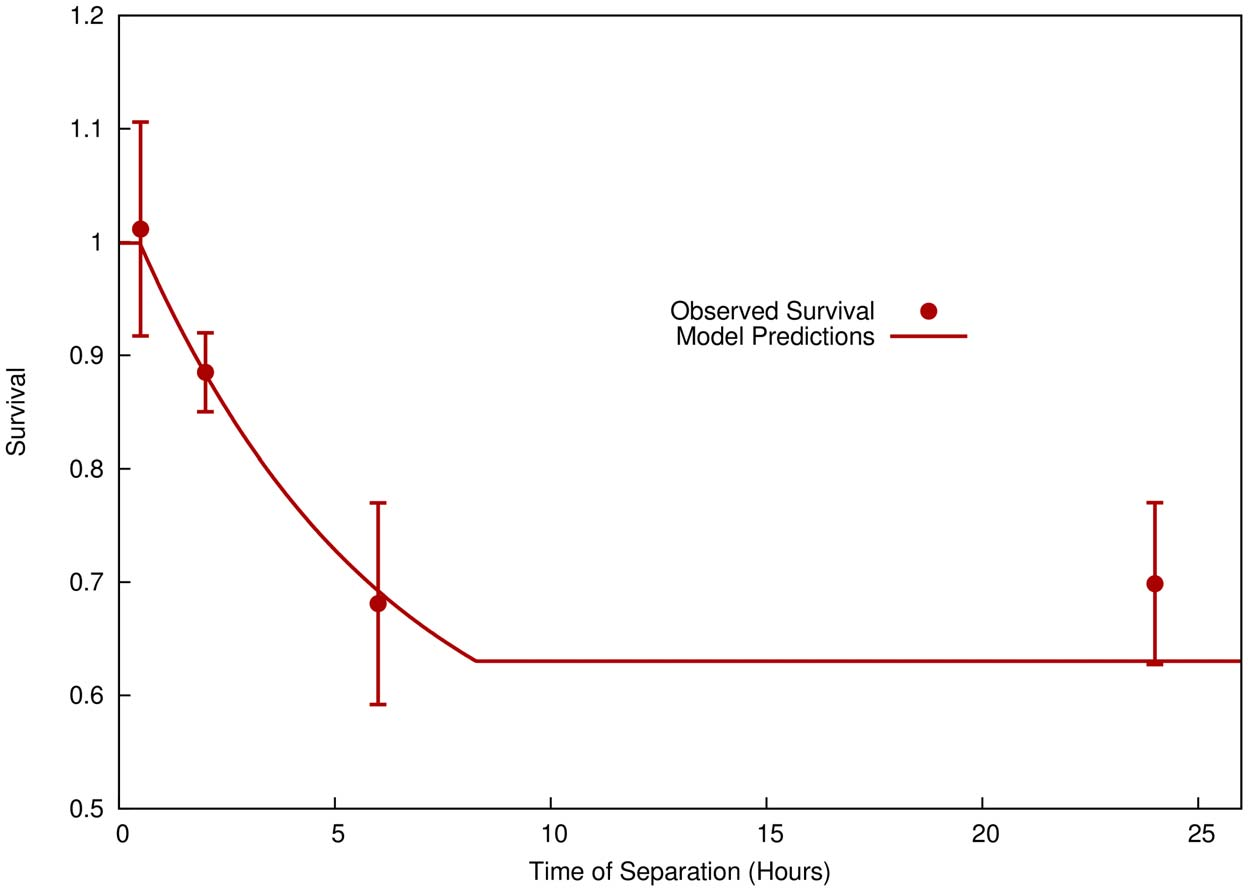
Effect of modulated field exposures when cells are separated after defined times. Survival data is presented for shielded DU145 cells which were incubated with exposed cells for a series of times, before being separated into separate flasks. A clear time-dependence is seen, on a relatively longer scale than that seen in media transfer experiments. A curve has been plotted based on the response parameters fitted to the experiments shown in Figure 3, showing good agreement between the kinetics assumed by the model and those observed experimentally.

Figure 5 shows the clonogenic survival of the non-irradiated population, showing that there is a clear temporal dependence of the signalling effect over a period of approximately 6 hours, substantially longer than that which is typically measured in media-transfer experiments, in line with the assumptions of this model. The predictions of the model based on the fit to the sparsely seeded clonogenic data is also presented, showing good agreement between the predicted and modelled signal kinetics.

### Skin Model

One of the major limitations of the above experimental studies is that no spatial information is provided because of the rapid propagation of signals through culture media. While robust measurements of cellular signalling in *in vivo* systems are not yet available, *in vitro* tissue models such as that of Belyakov et al [33] provide information on these effects in tissues, enabling the spatial kinetics of the model to be tested.

**Figure 6:**
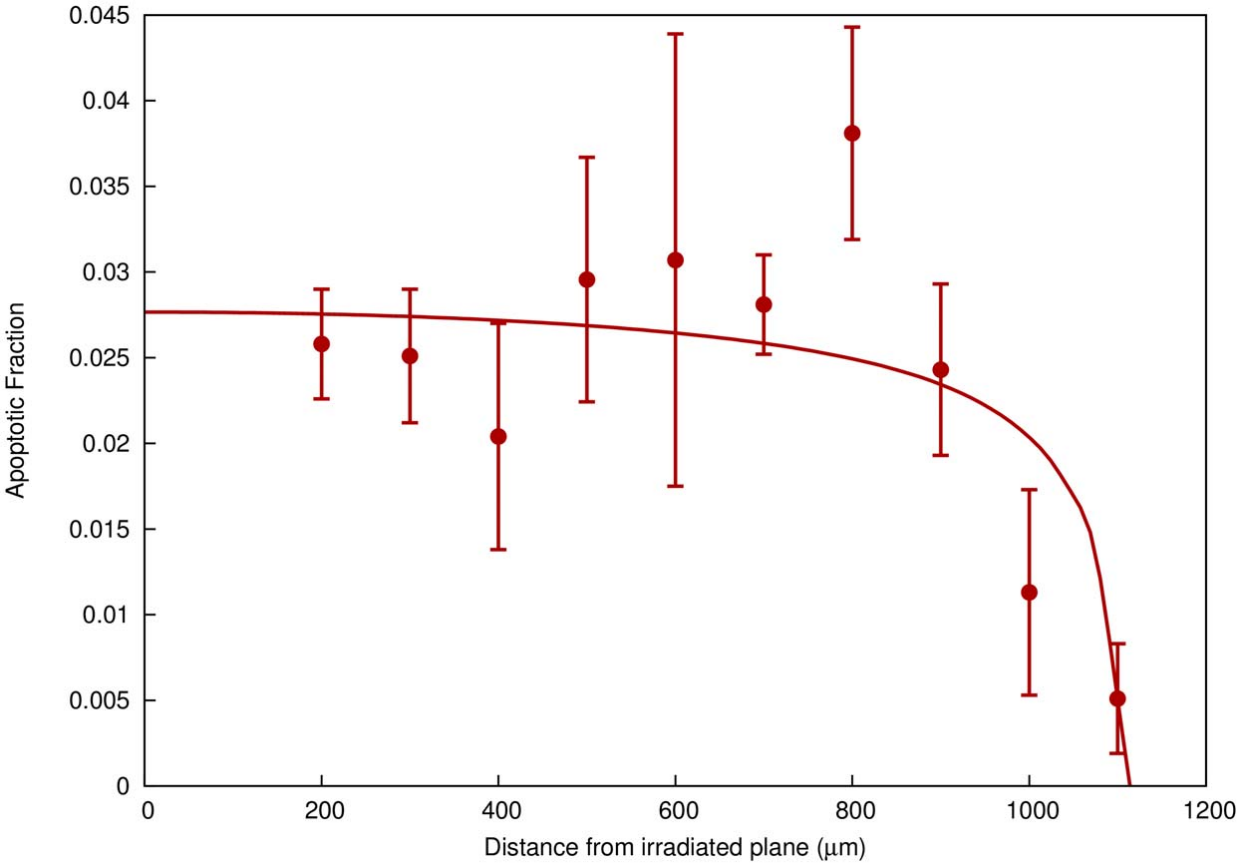
Spatial variation of cell death due to signalling effects in an in vitro skin model. Excess rates of apoptosis are plotted as a function of distance from a plane of cells irradiated with an α particle microbeam, compared to control cells in an unirradiated model. Significant increases in apoptosis are seen out to distances of more than 1 mm. A curve has been plotted showing the predictions of the model in this work, taking into account spatial propagation of the signal.

In Belyakov et al, a 3D human skin model was grown *in vitro*, comprising an 8 mm diameter cylinder of multiple cell layers, with a total thickness of approximately 75 µm. A diameter of this cylinder was irradiated using a 5 µm wide α particle microbeam, exposing a small, well-defined plane of cells to a dose of approximately 1 Gy. Levels of apoptosis were quantified throughout the cylinder at various distances from the irradiated slice, showing an increase in apoptosis when compared to unirradiated samples, as shown in Figure 6, which was attributed to intercellular communication.

In tissues of this type, signal molecules must propagate through the tissue structure, rather than growth media, which dramatically reduces their range and leads to a clear spatial dependency. To take this into account, signal diffusion was explicitly numerically modelled as a function of time and distance from the irradiated slice, as described in the supporting information. This allowed for the value of τ, and thus the probability of a bystander response, to be calculated as a function of distance from the irradiated plane.

The model was fit to the data by varying κ, γ, the apoptosis rate in cells which respond to the signals, and the signal range, which corresponds to 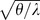 in this 1−D case. λ and ρ_t_/ρ_max_ were taken to have the same values as in the single-cell experiments outlined above.

As can be seen in Figure 6, the model accurately reproduces the observed behaviour as a function of position, with a largely flat region in the vicinity of the irradiated plane followed by a sharp fall. Signal kinetic and response parameters are presented in Tables 1 and 2, and the signal range obtained was 680±20 µm. This corresponds to a diffusion coefficient of 1.4×10^−6^ cm^2^ s^−1^, in line with diffusion coefficients of small molecules in cytoplasm [34].

This agreement indicates that the spatially-dependent formulation of the model is able to accurately describe the signal kinetics as a function of time and position in tissue structures, raising the possibility of a future generalisation of this model to full 3-D calculations in more clinically relevant scenarios.

## Discussion

There is now no doubt that non-targeted effects play a significant role in the *in vitro* survival of cells exposed to ionising radiation, as well as a range of other insults [35]–[37]. In this work we present a model of radiation response and the resulting signalling, which seeks to link data from many different classes of experiment to provide insights into the importance of intercellular signalling.

We have successfully modelled biological endpoints from a variety of studies, reproducing observed trends and obtaining quantitative agreement with reasonable sets of fitted parameters. Significantly, it provides a common description for effects observed in media-transfer, modulated field and tissue experiments, resolving many of the discrepancies observed between these different conditions and with currently published models.

The primary distinctions in this model are that signal production is taken to be a characteristic of the cell line, occurring for a time proportional to delivered dose, and that the response probability is related to the amount of time the signal is above a given threshold value, rather than total signal exposure. One important factor highlighted by these assumptions is that media transfer protocols, although commonly used, may systematically under-estimate the importance of intercellular communication, as cells only see the decaying signal concentration, rather than the prolonged exposure which occurs when cells are in continual contact.

Due to the inherently complex nature of these effects, there are a large number of cell-line and experiment dependent parameters which must be taken into account. However, based on the assumption of some common parameters (λ and ρ_t_), agreement was obtained across a range of experiments, with broadly comparable response parameters. Some discrepancies do exist, however – for example, fitted values of ν are significantly lower in the high-density media transfer experiments than in the modulated field experiments – suggesting it is still incomplete.

Several obvious future refinements are apparent. One present limitation is that many of the parameters are taken as exact across whole populations, which leads to a degree of sharpness which is not characteristic of most biological systems. In reality, many of these characteristics would have a spectrum of values, and the fitted values only represent the effective value, which may lead to discrepancies in certain conditions.

Additionally, the model is currently formulated in terms of single, acute radiation doses, as this is used in the experiments considered here. It would be valuable to extend the model to incorporate more information about cell repair and resulting temporal variations of signal production, to allow for its application to systems such as fractionated radiation exposures or extremely low dose-rate exposures, which are relevant to cancer risk and where exposure time is much longer than the timescale of the experiments considered here.

Finally, the nature of the signal itself and resulting response is not yet explicitly incorporated. While a single concentration is used in this model, a variety of biological factors including cytokines, signalling molecules and reactive species have been implicated in these processes [9]. Although analysis of signalling in a skin model suggests the effect is limited by the transport of a molecule with molecular weights on the order of hundreds of atomic mass units (Daltons), it is unclear if this diffusion rate would translate to more general *in vivo* situations, due to variations in tissue structure, vasculature, and so forth. Similarly, the origin of this signal is also not considered. One possibility is that it is produced as a consequence of DNA damage and repair, as this is a common theme, not only in intercellular communication effects observed due to radiation, but also in other systems, such as UV irradiation [35], heat shock [37], or exposure to chemotherapeutic drugs [36].

This may also explain the observed time-dependence of the signal in this work, as the time-scale associated with signal production (represented by γ values of 1–3 hours per Gy, corresponding to signal production times ranging from approximately 1 to 24 hours in the conditions considered here) is similar to that typically associated with DNA double strand break repair (typically described as including a fast component, with a repair half-time on the order of 30 minutes, and a slow component, with a half-time on the order of hours), suggesting a possible link between these processes [38], [39].

One area in which investigation of the above areas would be valuable is the possibility that these effects are driven by an ensemble of signals, some of which may have protective or even proliferative effects, for which there is now some evidence [40]. While some of the apparent protective effects may be explained in the context of this model as reductions in the strength of intercellular signalling effects in irradiated populations, the presence of an additional, proliferative, signal may be needed to fully explain some of these results.

However, despite these limitations, the model has proven to be able to robustly describe a variety of experimental conditions, suggesting it can provide useful insights into these mechanisms. One of the most significant implications of this model is that (as noted in previous work [21]) despite being commonly described as “bystander” effects, intercellular communication contributes significantly to the survival of all cells in a population, even those directly exposed to radiation, being perhaps the dominant source of cell death up to doses of several Gray (Figure S3).

Although this effect was first clearly described in unirradiated populations, the name “bystander effect” may eventually prove to be a misnomer. Instead, there is the suggestion that the underlying mechanism may not only be involved in long-range signalling between irradiated and unirradiated populations, but also be involved in a much wider range of conditions, potentially including paracrine signalling within an exposed population (the effects of which have been termed “cohort effects” [10]). Significantly, this implies that many of the effects of intercellular signalling are already implicitly incorporated in empirical measurements at higher doses (e.g. Cancer risks from environmental or therapeutic exposures) which are often interpreted primarily as being due to “direct” effects.

Furthermore, if the significant contribution of intercellular signalling to cell killing in *in vitro* modulated exposures is representative of its contribution to *in vivo* exposures - as would be expected because cells in an organism are, by definition, in contact for extended periods – then these signalling processes may well act as a biological threshold for dose conformation in radiation therapy. Even signal ranges on the order of the 1 mm estimated from the skin model would have the potential to lead to a spatial variation in survival which is not well represented by the variation in dose, and potentially mitigate many of the benefits of improved dose delivery techniques. An obvious application of this model is to carry out full spatially- and temporally-dependent calculations of intercellular signalling using clinically relevant structures and dose plans, in conjunction with models of cellular survival [21], [31], to determine the possible impact of these contributions. While this 3-D generalisation is mathematically straightforward, *in vivo* measurements of these effects would be valuable to provide a test for these predictions.

In conclusion, we have developed a novel model of intercellular signalling following radiation exposure, incorporating signal generation, genotoxic responses and the effects of direct irradiation. This model was tested against a variety of systems and endpoints, showing good agreement, including in *in vitro* tissue structures. This model highlights the potential significance of intercellular communication in biological responses to ionising radiation, particularly in systems where irradiated and non-irradiated cells remain in contact for extended periods of time. If validated *in vivo*, this model would significantly impact the interpretation of many factors in radiation biology, suggesting a move away from the concept of purely local doses towards models explicitly incorporating intercellular signalling.

## Supporting Information

Figure S1 **Numerical model of kinetics of signals following irradiation.** The diffusion of signals from populations of irradiated cells was modelled numerically as described in the text, for a variety of cell densities. Signal intensities were plotted either as total signal level (left) or as a signal normalised to the level at saturation for that cell line (right). It can be seen that although the total signal level varies by several orders of magnitude as the cell density is increased, the rate at which the signal approaches saturation is much less variable.
(TIFF)

Figure S2
**Maximum signal concentration and production rate.** Models of signal production as illustrated in Figure S1 have been characterised in terms of the maximum signal concentration (left) and their effective rate parameter (right). The maximum signal concentration as a function of cell number has been fitted to the predictions of the analytic approximation used in this work, showing good agreement. The effective rate constant λ_eff_ has been fit with a function of the form 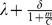 where C is the total cell number, λ is the signal decay rate, and δ and m are fitting parameters. It can be seen that the range of effective rate constants is small, reaching less than 3 times the signal decay rate.(TIFF)

Figure S3 **Contribution of intercellular signalling to cell killing.** Survival was calculated for uniformly exposed cells using parameter sets fitted to observed results and for the same cell line without signalling effects. These values were then used to calculate the fraction of cell killing due to intercellular communication, as a function of dose, plotted above. It can be seen that at clinically used doses (typically 2 to 4 Gray), these effects are responsible for a large fraction of cell killing, and that this contribution is strongly cell-line dependent.
(TIFF)

Models and Methods S1 **Detailed information on numerical implementations of signal propagation and comparisons with analytic descriptions used in the main text.**
(PDF)

Example Code S1 **Basic python implementation of model for an idealised half-field irradiation, of the kind used by Butterworth et al.** Currently implements parameters for DU145 cells, generating in- and out-of-field survival for a series of dose levels, as used to fit the data in Figure 3.
(PY) Click here for additional data file.
