# Supplementary figures and images for: A Kinetic-Based Model of Radiation-Induced Intercellular Signalling
Source: PLoS One. 2013 Jan 22;8(1):e54526. doi: 10.1371/journal.pone.0054526 (PMC3551852; doi:10.1371/journal.pone.0054526)

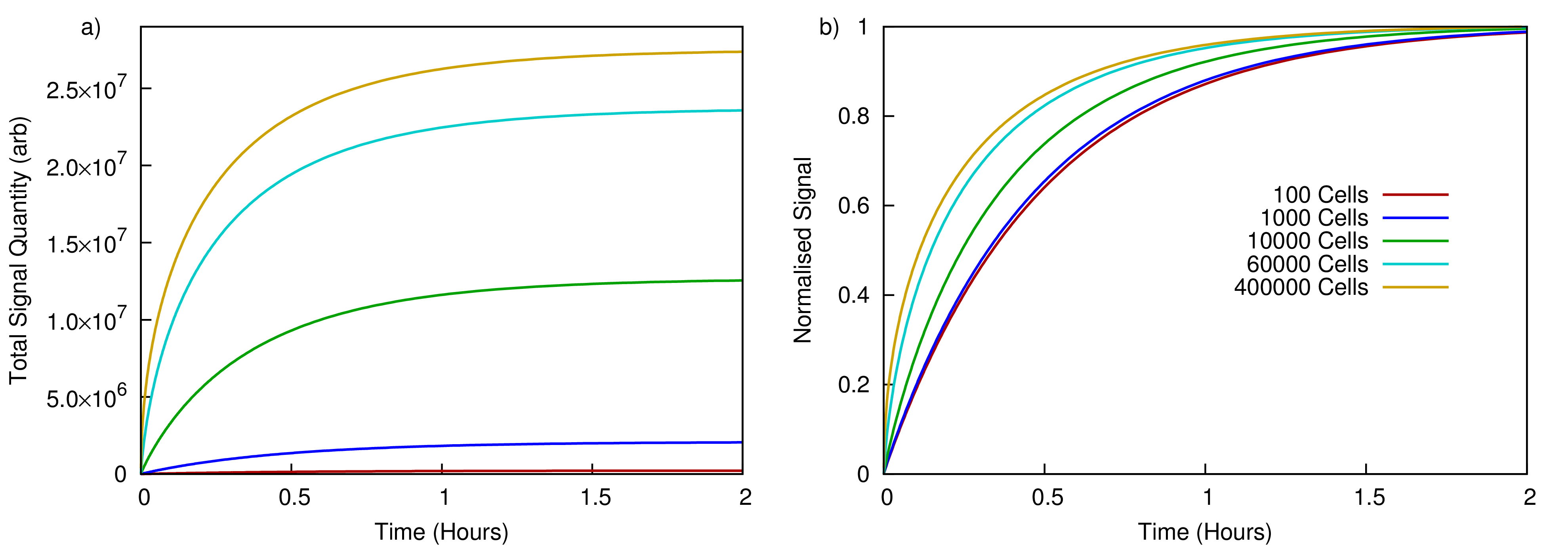

Supplement: Figure S1 — Numerical model of kinetics of signals following irradiation. The diffusion of signals from populations of irradiated cells was modelled numerically as described in the text, for a variety of cell densities. Signal intensities were plotted either as total signal level (left) or as a signal normalised to the level at saturation for that cell line (right). It can be seen that although the total signal level varies by several orders of magnitude as the cell density is increased, the rate at which the signal approaches saturation is much less variable. (TIFF) [file pone.0054526.s001.tiff]

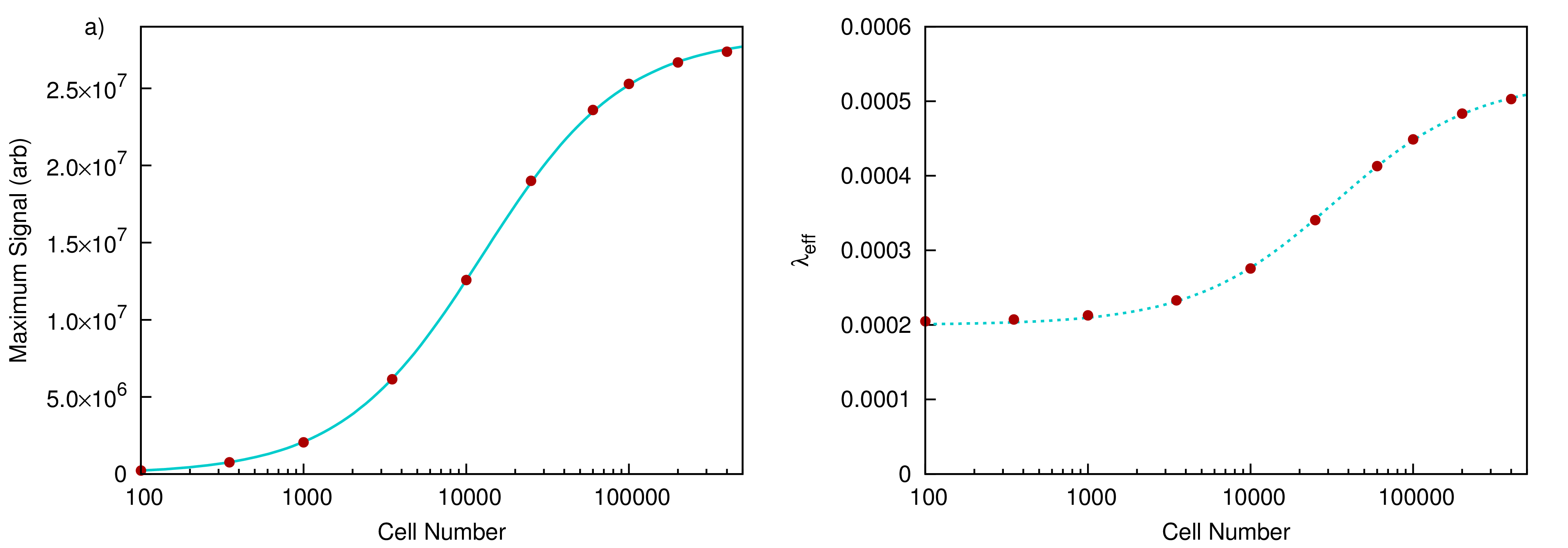

Supplement: Figure S2 — Maximum signal concentration and production rate. Models of signal production as illustrated in Figure S1 have been characterised in terms of the maximum signal concentration (left) and their effective rate parameter (right). The maximum signal concentration as a function of cell number has been fitted to the predictions of the analytic approximation used in this work, showing good agreement. The effective rate constant λeff has been fit with a function of the form where C is the total cell number, λ is the signal decay rate, and δ and m are fitting parameters. It can be seen that the range of effective rate constants is small, reaching less than 3 times the signal decay rate. (TIFF) [file pone.0054526.s002.tiff]

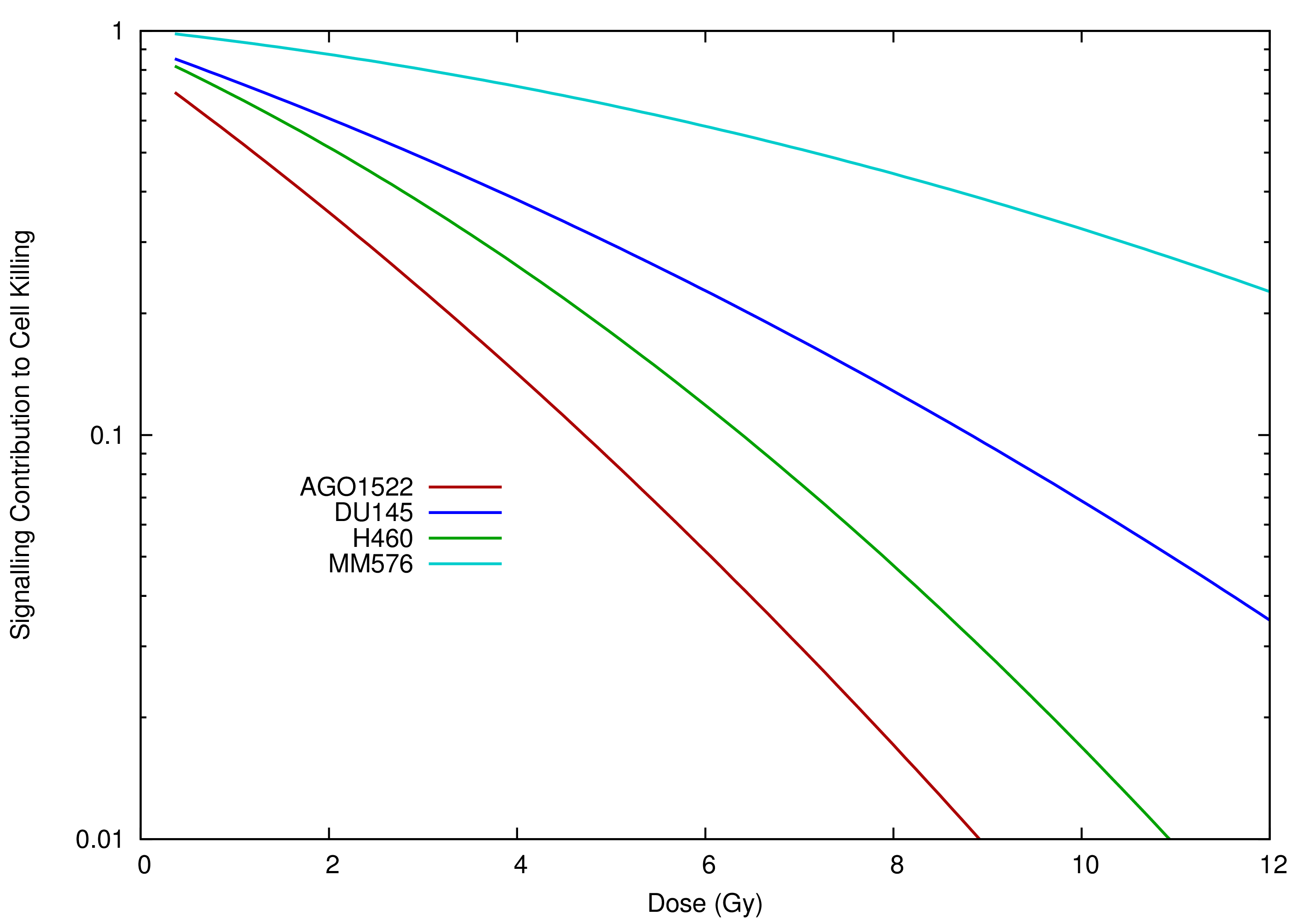

Supplement: Figure S3 — Contribution of intercellular signalling to cell killing. Survival was calculated for uniformly exposed cells using parameter sets fitted to observed results and for the same cell line without signalling effects. These values were then used to calculate the fraction of cell killing due to intercellular communication, as a function of dose, plotted above. It can be seen that at clinically used doses (typically 2 to 4 Gray), these effects are responsible for a large fraction of cell killing, and that this contribution is strongly cell-line dependent. (TIFF) [file pone.0054526.s003.tiff]
