# Supplementary material for: A Kinetic-Based Model of Radiation-Induced Intercellular Signalling
Source: PLoS One. 2013 Jan 22;8(1):e54526. doi: 10.1371/journal.pone.0054526 (PMC3551852; doi:10.1371/journal.pone.0054526)
Supplement: Models and Methods S1 — Detailed information on numerical implementations of signal propagation and comparisons with analytic descriptions used in the main text. (PDF) [file pone.0054526.s004.pdf]

A Kinetic-based Model of Radiation-Induced Intercellular Signalling

Stephen J McMahon, Karl T Butterworth, Colman Trainor, Conor K McGarry, Joe M O'Sullivan, Giuseppe Schettino, Alan R Hounsell, Kevin M Prise

Supplementary Information

Signal Propagation - Analytic

As described in the main text, a signal which propagates throughout the media by diffusion while simultaneously decaying can be described as

$$\frac{\partial \rho(\underline{r},t)}{\partial t} = \theta \nabla^2 \rho(\underline{r},t) - \lambda \rho(\underline{r},t) \tag{1}$$

where $\rho(\underline{r},t)$ is the signal concentration as a function of position, $\underline{r}$, and time t; θ is the diffusion coefficient; and λ is the signal decay coefficient.

However, the more relevant system in which there are irradiated cells which are actively signalling within the volume requires the addition of sources of signal to describe the system. A general rate equation for this system for a series of point signalling cells at positions $\underline{r}_0$, $\underline{r}_1$, ... would be given by

$$\frac{\partial \rho(\underline{r},t)}{\partial t} = \theta \nabla^2 \rho(\underline{r},t) - \lambda \rho(\underline{r},t) + \sum_i \delta_{\underline{r},\underline{r}_i} \eta \frac{\rho_{max} - \rho(\underline{r},t)}{\rho_{max}} \tag{2}$$

where δ is the Kronecker delta function; η is the signal produced per cell; and the sum is carried out over all of the signalling cells. As noted in the main text, it is assumed that cells seek to maintain some equilibrium concentration ρ_{max}, so the signal produced per cell is related to the difference between the local concentration and this maximum value.

Even in the case of a single cell producing signal, equation S.2 does not have an analytic solution, and approximations are significantly complicated due to the variation in potential cell distributions, boundary conditions due to flask volume, and interplay between the diffusion and decay coefficients.

Some general observations about the signal kinetics in this system can be made by considering the 1 dimensional case, however. This can be viewed as corresponding to, for example, a confluent or near-confluent cell monolayer generating signal which fills the remainder of the flask, or an irradiated plane of cells in a skin model. In this case, the signal is taken to only vary in the z direction, and the rate equation becomes

$$\frac{\partial \rho}{\partial t} = \theta \frac{\partial^2 \rho}{\partial z^2} - \lambda \rho$$

which gives an equilibrium condition of

$$\lambda \rho = \theta \frac{\partial^2 \rho}{\partial z^2}$$

This equation can easily be solved, with a general solution of:

$$\rho = C_1 e^{z\sqrt{\frac{\lambda}{\theta}}} + C_2 e^{-z\sqrt{\frac{\lambda}{\theta}}}$$

where C_1 and C_2 are fitting constants. If the irradiated cell layer at $z = 0$ are sufficient to maintain a concentration of ρ_{max}, then $C_1 + C_2 = \rho_{max}$. The ratio between C_1 and C_2 then depends on the remaining boundary condition. The most common case is when there is a maximum depth z_{max} past which the signal cannot diffuse (corresponding, for example, to the depth of the media, or the thickness of the skin slice). In the limit where where this depth z_{max} is much larger than $\sqrt{\frac{\lambda}{\theta}}$, this becomes a simple exponential decay, $\rho = \rho_{max} e^{-z\sqrt{\frac{\lambda}{\theta}}}$.

This illustrates the idealised equilibrium behaviour of a plane of signalling cells, maintaining a concentration of ρ_{max} locally which falls off exponentially with increasing distance, with a range given by $\sqrt{\frac{\lambda}{\theta}}$. In practice, as z_{max} is finite, this fall-off is somewhat slower than a pure exponential, as $\frac{\partial\rho}{\partial z}$ must go to zero at this boundary, requiring a non-zero value of C_1.

The range value of $\sqrt{\frac{\lambda}{\theta}}$ is of some interest, as this provides some insight into the relative values of the diffusion and decay coefficients. There is little evidence in the literature of spatial variation of responses following modulated field exposures in in vitro cultures of sparsely seeded cells, which suggests that the range is at least on the order of the size of a typical cell culture flask. However, if, for example, the decay coefficient had a value of 0.01 min^{-1} (similar to values suggested in other work), a range of 1 cm implies a diffusion coefficient on the order of 2×10^{-4} cm^2/s, which is significantly greater than that of proteins which are often implicated in these signalling processes (on the order of 2×10^{-6} cm^2/s).

This suggests either the involvement of an extremely light signalling molecule (individual radicals or ions such as Ca^+, NO• and OH•) or that the signal disperses by a mechanism which is not purely diffusion-driven. This latter observation is likely in the general case of media in cell culture flasks, media is often disturbed in several ways during these experiments (due to handling, variations in temperature, and so forth), which can lead to a significant increase in the rate of dispersion of signalling molecules. Despite the likely contribution of other dispersive processes, a diffusion-like description would still be expected to be a useful model of the the how the signal concentration evolves in solution, provided the true process is also reasonably spatially homogeneous.

Signal Generation - Numerical

More general solutions similar to the above 1 dimensional approach are not applicable in 3 dimensions, even for a single source cell. However, such systems can be simply numerically modelled, allowing for full temporal and spatial information to be obtained, albeit at the expense of computational time.

To illustrate this, a simple numerical model was developed. Briefly, as noted above the diffusion equation can be expressed as

$$\frac{\partial\rho}{\partial t} = \theta\frac{\partial^2\rho}{\partial z^2} - \lambda\rho$$

in 1 dimension. If space is discretised into a series of voxels, z_i of width Δz, then we can say

$$\left.\frac{\partial^2\rho}{\partial z^2}\right|_{t,z_i} = \frac{\rho_{t,z_{i-1}} - 2\rho_{t,z_i} + \rho_{t,z_{i+1}}}{\Delta z^2}$$

and express the time evolution as

$$\left.\frac{\partial\rho}{\partial t}\right|_{t,z_i} = \frac{\theta}{\Delta z^2}\left(\rho_{t,z_{i-1}} - 2\rho_{t,z_i} + \rho_{t,z_{i+1}}\right) - \lambda\rho_{t,z_i}$$

$$\rho_{t+\Delta t,z_i} = \rho_{t,z_i} + \Delta t\left.\frac{\partial\rho}{\partial t}\right|_{t,z_i}$$

where Δt is the time-step being used in the simulation. The above equations can be trivially numerically implemented and used to simulate the time evolution of the signal produced by a plane of signalling cells.

For the skin model fitted in this work, the above set of equations were used to describe the time evolution of the system. The tissue volume was approximated as a series of 25 µm thick voxels with associated media concentrations, spanning a total thickness of 3 mm (where signal levels were ~1% of that at $z = 0$). Boundary conditions were set such that there was no flow of signal out of the tissue model at either end of the structure.

To allow calculation of the evolution of the signal with time following irradiation, at time $t = 0$, the signal concentration at $z = 0$ was set to ρ_{max}, and the remaining cells were allowed to evolve according to the above equations. This was modelled until a time γD, when the cells at $\rho = 0$ were assumed to cease signalling, and the concentration there was allowed to evolve freely, as in the other voxels. To calculate the probability of a response, the total time for which cells were exposed to signals greater than a value of ρ_t was recorded at each point.

While calculations of this type are tractable for this skin model, thanks to its small size and the 1 dimensional approximation, full calculations of the 3 dimensional diffusion in modulated exposure of sparsely seeded cells are computationally prohibitive. However, some example calculations can be carried out, to test the validity of the analytic approximations presented in the Methods.

Briefly, the media volume is divided up into a 3 dimensional array of voxels, each with a volume approximately corresponding to that of a single cell (10 µm a side). The equations outlined above for the 1-D case can then trivially be generalised to three dimensions, by noting that the three dimensional Laplacian is given by

$$\nabla^2\rho = \frac{\partial^2\rho}{\partial x^2} + \frac{\partial^2\rho}{\partial y^2} + \frac{\partial^2\rho}{\partial z^2}$$

where each partial spatial derivative can be approximated as above. At each time step, voxels which contain signalling cells have their signal concentration set to ρ_{max}, and the signal evolution is calculated according to the above equations.

As most experiments are based on the irradiation of cells sparsely seeded on the base of a flask, this was taken as a test scenario. A 1000×1000×500 array of voxels (corresponding to a 1 cm × 1 cm × 0.5 cm volume, or 0.5 mL) was initialised with varying numbers of cells plated on the bottom plane, and allowed to evolve over time. In this model, λ was set to 0.01 min^{-1}, θ to 0.005 mm^2 s^{-1}, and the time was stepped in 1 second intervals. Figure S1 presents the average signal concentration as a function of time in this volume, for a range of cell densities.

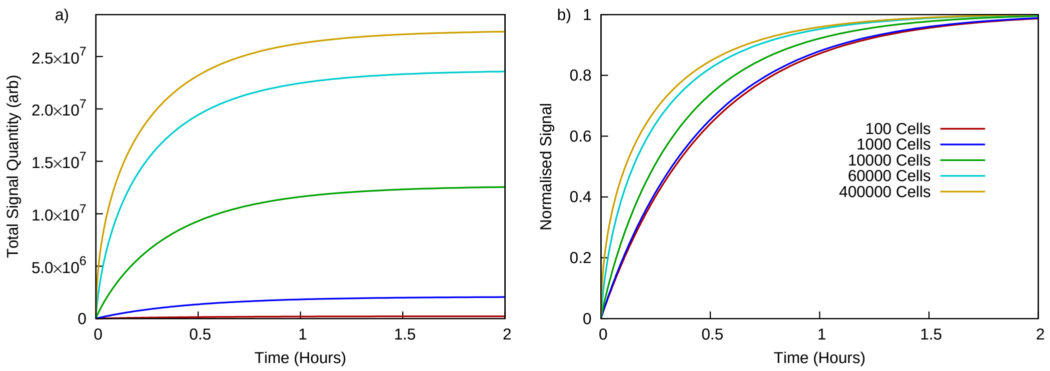

Supplementary Figure S 1: Numerical model of kinetics of signals following irradiation. The diffusion of signals from populations of irradiated cells were modelled numerically as described in the text, for a variety of cell densities. Signal intensities were plotted either as total signal level (left) or as a signal normalised to the level at saturation for that cell line (right). It can be seen that although the total signal level varies by several orders of magnitude as the cell density is increased, the rate at which the signal approaches saturation is much less variable.

It can be seen that, in all cases, the signal kinetics are broadly similar with the analytic model presented in the main text. At low concentrations, this agreement is close to exact, with the initial build-up being almost exactly a simple exponential, with a rate coefficient equal to λ. At higher cell numbers, however, this begins to break down somewhat, as the build-up speed increases, but the curve also becomes sub-exponential, slowing significantly as the signal concentration approaches saturation.

Two key characteristics were investigated: the maximum signal concentration, and the rate at which the signal builds up. The variation of these parameters are shown in Figure S2, compared to what is predicted based on the analytic model used in the main text. The variation of the maximum signal concentration with cell number is compared to the function $\frac{\rho_{max}}{1+\lambda V/\nu C}$, where the combined value $\nu/\lambda V$ is fitted as an empirical parameter. It can be seen that there is good agreement between the analytic and numerical predictions of the maximum signal concentration.

The variation of the rate of signal build-up is more complex, as at higher cell densities the rate of build-up cannot be described as a simple exponential, making direct comparisons to the analytic rate parameter λ difficult. As a compromise, a value λ_{eff} has been defined as 1 divided by the time taken for the signal to build up to 63.2% of its maximum value (which is equal to λ in the case of a pure exponential function). This value is plotted in figure S2. It can be seen that the decay coefficient λ represents the minimum signal build-up rate, and although the number of cells varies by five orders of magnitude in these models, the effective signal build-up rate varies by only a factor of 2.6 over this range. This variation is dramatically less than the $\lambda + \nu C$ variation predicted by the simple formulation of the analytic model. Thus, as noted in the text, an approximation is made, fixing the build-up rate to the decay coefficient, λ. This gives good agreement over the majority of conditions considered here, and leads to only minor discrepancies in the total signal production time at high cell densities (on the order of ten minutes), which are likely offset by other biological factors (such as a delay between irradiation and the generation of signal, for example).

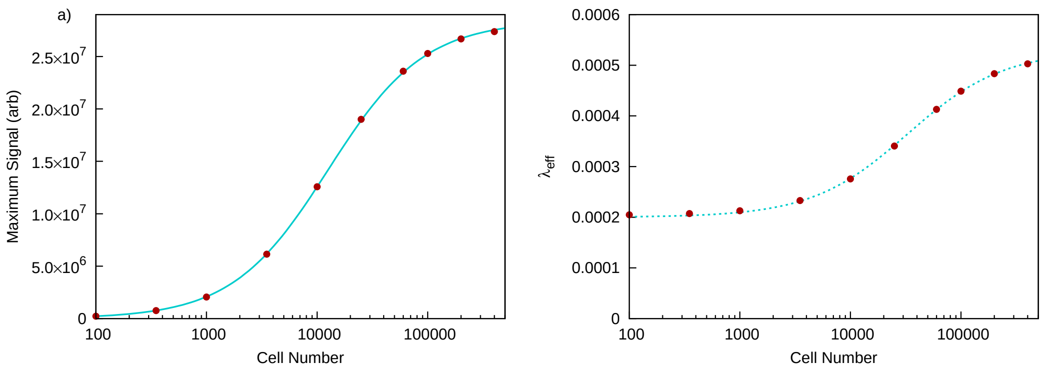

Supplementary Figure S 2: Maximum signal concentration and production rate. Models of signal production as illustrated in Figure S1 have been characterised in terms of the maximum signal concentration (left) and their effective rate parameter (right). The maximum signal concentration as a function of cell number has been fitted to the predictions of the analytic approximation used in this work, showing good agreement. The effective rate constant λ_{eff} has been fit with a function of the form $\lambda + \delta/(1+m/c)$ where C is the total cell number, λ is the signal decay rate, and δ and m are fitting parameters. It can be seen that the range of effective rate constants is small, reaching less than 3 times the signal decay rate.

Overall, these results suggest that the analytic approximation outlined in the main text is a good approximation of the more realistic kinetics of the system, supporting its use to facilitate the fitting of parameters to describe the observed experimental results. It also shows that further generalisations to describe more physically meaningful 3-D volumes can trivially be achieved, providing a basis for future work investigating the implications of intercellular signalling in realistic treatment volumes.

Radiation-Induced Signalling Contribution in Uniform Fields

As noted in the main text, one of the main assumptions of this model is that responses to intercellular signalling effects can occur in all cells, regardless of the level of direct radiation exposure they experience. This implies that these processes may contribute significantly to cell killing even in uniform exposures.

To quantify the possible contributions of this effect in uniform exposures, cellular survival in two scenarios can be compared: Firstly, for uniformly irradiated, densely seeded (such that $Cv \gg \lambda V$) cell cultures, based on the parameters presented in Tables 1 and 2; secondly, an identical set-up was modelled, but with signalling removed (by setting $\gamma = 0$). By comparing the survival in each case, the fraction of cell killing which is due to signalling processes can be calculated. These calculations were carried out, as a function of dose, for each of the cell lines for which modulated field data was modelled (AGO1522, DU145, H460 and MM576 cells). The resulting calculated contributions are plotted in Figure S3.

It can be seen that, across all cell lines, intercellular signalling processes contribute the majority of cell killing at low doses, and more than 30% of cell killing at the 2 Gy doses typically used in fractionated radiotherapy in all lines. There is also considerable differences between different cell lines in the variation of the intercellular communication contribution - with it being much more rapidly diminished in more radio-sensitive cell lines than in radio-resistant cell lines, due to the

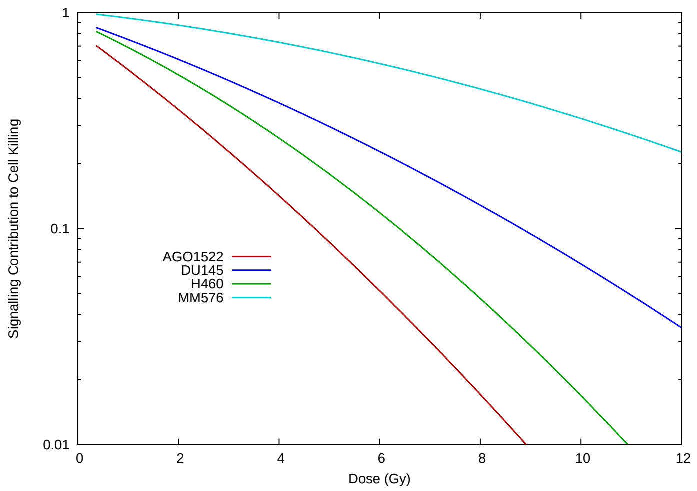

Supplementary Figure S 3: Contribution of intercellular signalling to cell killing. Survival was calculated for uniformly exposed cells using parameter sets fitted to observed results and for the same cell line without signalling effects. These values were then used to calculate the fraction of cell killing due to intercellular communication, as a function of dose, plotted above. It can be seen that at clinically used doses (typically 2 to 4 Gray), these effects are responsible for a large fraction of cell killing, and that this contribution is strongly cell-line dependent.

relatively greater contribution of direct damage. Notably, in the most radio-resistant MM576 cell line, even at doses of 12 Gray, approximately 20% of cell killing is still due to signalling effects, rather than direct radiation effects. This is a dramatically different characterisation of the role of intercellular signalling than that seen in most models, where it is typically described as a primarily low-dose phenomenon, and suggests very different interpretations of many radiobiological phenomena than the traditional direct damage models of radiation exposure.
